# Identifying Ecological Corridors and Networks in Mountainous Areas

**DOI:** 10.3390/ijerph18094797

**Published:** 2021-04-30

**Authors:** Di Zhou, Wei Song

**Affiliations:** 1 Key Laboratory of Land Surface Pattern and Simulation, Institute of Geographic Sciences and Natural Resources Research, Chinese Academy of Sciences, Beijing 100101, China; zd15071032867@163.com; 2 School of Geosciences, Yangtze University, Wuhan 430100, China

**Keywords:** ecological corridor, ecological network, landscape pattern, Linkage Mapper software, Chongqing, China

## Abstract

Since the 1950s, human activities have been driving economic development and land changes, hindering the conservation of biological habitats and landscape connectivity. Constructing ecological networks is an effective means to avoid habitat destruction and fragmentation. Mountain areas are hotspots of biological habitats and biodiversity; however, the pace of urbanization in mountain areas is also accelerating. To protect an ecosystem more effectively, it is necessary to identify ecological corridors and ecological networks. Therefore, based on the Minimal Cumulative Resistance model and taking Chongqing in China as an example, the identification of potential ecological corridors and the construction of an ecological network in Chongqing were realized using the Linkage Mapper software. The results were as follows: (1) From 2005 to 2015, the patch area of cultivated land and grassland in Chongqing decreased by 0.08% and 1.46%, respectively, while that of built-up areas increased by 1.5%. The fragmentation degree of cultivated land was higher, and the internal connectivity of forestry areas was worse. (2) In total, 24 ecological sources were selected, and 87 potential ecological corridors and 35 ecological nodes were generated using the Morphological Spatial Pattern Analysis and the Conefor2.6 software. The total length of the ecological network in Chongqing is 2524.34 km, with an average corridor length of 29.02 km. (3) The overall complexity and network efficiency are high, but the spatial distribution of ecological corridors is uneven, especially in the southwest of Chongqing.

## 1. Introduction

Currently, the global scale of cities continues to grow [1,2], with an expanding scope of human activities [3], putting the global biodiversity at risk of further degradation [4,5]. Although considerable efforts have been made by governments around the world to protect biodiversity [6,7,8,9,10], the coordination of economic development and biodiversity conservation is still challenging [11,12]. Among the factors affecting biodiversity, fragmentation and loss of biological habitat are considered the most important ones [13]. As an ecological network can adequately depict the ecological processes [14] and can protect habitats and maintain landscape connectivity [15,16], the construction of ecological networks has become a research hotspot in ecosystem protection [17,18].

An ecological network is a complex network having ecological corridors and ecological nodes as its constituent elements that connect core areas, nature reserves, and other landscape elements [19,20,21,22]. Ecological networks have first been used in the study of biological protection [23]. Along with the expansion and improvement of ecological network functions, they are widely used in biodiversity conservation [21]. For example, from the 1970s to the 1990s, some Eastern European countries, the Netherlands, Canada, and the United States developed network plans for nature reserves [24] and began to emphasize the role of ecological interconnection. In the early 21st century, Europe established an ecological network framework that emphasized green protection [23]. In the Convention on Biological Diversity 2010, the Aichi Target 11 (Aichi refers to Japan’s Aichi Prefecture) defined the goal of ecosystem protection by 2020, which is to protect at least 17% of the land and inland water areas and 10% of the marine area by linking ecological areas [10,25]. It further highlights the significance of using ecological networks for biodiversity conservation.

An ecological corridor is an important tool connecting ecological networks [26]. Ecological corridors were originally designed to connect natural habitats for wildlife protection [23]. However, due to the interference from human activities [27], habitat fragmentation around the world has become increasingly prominent [28], and against this background, in 1975, Wilson and Willis put forward the view of connecting broken patches with corridors to weaken the impact of habitat fragmentation [23]. The concept of ecological corridors was put forward for the first time in the early 21st century by Jordan, based on reliability theory [29]. Subsequently, scholars have engaged in extensive discussions and studies on ecological corridors. For example, Bowers and McKnight put forward the necessity of constructing ecological corridors in North America [30], and Chettri et al. discussed the importance of constructing ecological corridors in the Kangchenjunga landscape from the view of maintaining forest ecosystems and protecting biodiversity [31].

Although the models and methods of describing ecological corridors and ecological networks are slightly different, the construction of an ecological network generally includes three steps [25,32]: (1) selecting the ecological source; (2) determining the resistance surface; (3) extracting ecological corridors and nodes. Based on these steps, the ecological network is constructed. The early selection of ecological sources was mostly limited to large landscape patches such as nature reserves and scenic spots [33,34], lending this method a certain subjectivity. With the development of relevant research methods, MSPA (Morphological Spatial Pattern Analysis) and other methods are gradually used to quantitatively evaluate the importance of ecological sources and landscape connectivity [4,35], thereby improving the scientific nature of ecological source selection. The resistance surface reflects the resistance of species passing through ecological patches during migration, which is one of the important factors to measure the risk of species migration. Currently, most scholars prefer to choose terrain conditions, human activity intensity [36], and land use type [37], among others, as the factors impacting the resistance surface, and some prefer to build the resistance surface based on land use types [35]. An ecological corridor is an important tool for the formation of an ecological network, and the research methods are constantly being improved. For example, Pomianowski and Solon used the GraphScape software to generate an ecological corridor [38], whereas Guo et al. constructed an ecological corridor based on the least-cost distance (LCD) and the least-cost path (LCP) [39]. Peng et al. combined the circuit theory and the Linkage Mapper software to identify the ecological corridor [40].

Ecological nodes are generally located at the convergence between the least-cost paths of the ecological corridors, at the site of the functional weakness area to connect scattered and isolated patches, which is the key to enhance the connectivity of ecological sources and promote the operation of ecological flows among ecological networks. The ecological resistance surface model is widely used in the extraction of ecological nodes [20]. Considering the resistance factors, the *MCR* (Minimal Cumulative Resistance) model is widely used in ecological corridor identification and ecological network construction [36]. For example, it has been used by Yang et al. [26] to identify the ecological corridor of Wuhan City, China, with the aim to construct an ecological network of urban agglomeration. Similarly, Dai et al. [41] combined the *MCR* model and the DOI (Duranton and Overman Index) to construct the ecological network of the Poyang Lake urban agglomeration in China.

Overall, current studies on ecological networks mainly focus on the construction of an urban agglomeration ecological network [42], a wetland ecological network, and a desert oasis ecological network [20], whereas studies in mountain areas are scarce [31,43]. In China, mountain areas account for 70% of the country’s terrestrial area [44], and ecosystem protection in mountain areas plays an important role for ecosystem protection in China [45]. In this context, this paper takes Chongqing, China’s “Mountain City”, as the research area, discussing ecological corridor identification and ecological network construction in mountain areas. The specific objectives are as follows: (1) to reveal the landscape pattern evolution of Chongqing from 2005 to 2015; (2) to use the Linkage Mapper software (The Nature Conservancy, Seattle, WA, USA) to identify potential ecological corridors in Chongqing; (3) in combination with GIS (Geographic Information System), to construct the potential ecological network of Chongqing.

## 2. Study Area and Data Sources

### 2.1. Study Area

**Figure 1:**
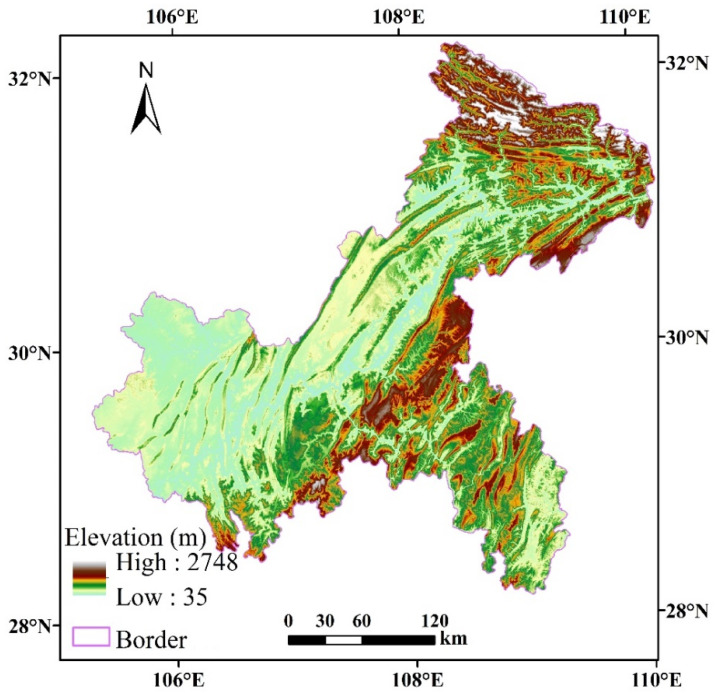
Geographical location of Chongqing, China.

The city of Chongqing (28°10′ N~32°13′ N, 105°11′ E~110°11′ E) is located in the southwest of inland China (Figure 1) and is the sole municipality in central and western China. It stretches over 470 km from east to west and 450 km from north to south, covering an area of 82,400 square kilometers. The terrain of Chongqing decreases from north and south in the direction of the Yangtze River valley; the area is mainly covered by mountains and hills, also known as the “Mountain City”. Chongqing is located in the Subtropical Zone, with abundant precipitation, four distinct seasons, and a high annual relative humidity. Driven by China’s reform and opening up, urbanization in Chongqing is rapid [46], with a rate above 81.04% in 2017 [47]. The gross domestic product in 2019 amounted to 236.06 billion RMB. The surge of the urban population and the rapid economic development result in an increased land development [48], with conflicts between humans and the ecological environment becoming increasingly prominent [49], resulting in a continuous deterioration of ecosystems [50].

### 2.2. Data Sources

We used four data types, namely land use data, DEM (Digital Elevation Model data), road data, and river data. Land use data were derived from the Resources and Environment Science and Data Center of the Chinese Academy of Sciences [51]. We selected data from the years 2005 and 2015, with a spatial resolution of 1 km; the data type was raster data. The main types of land use data included 6 first-class categories (cultivated land, forest land, grassland, water area, built-up areas, and unused land) and 17 s-class categories (paddy fields, dry land, forested land, shrub land, sparse forest land, other forestry areas, high-coverage grassland, medium-coverage grassland, low-coverage grassland, river channel, lake, reservoir pit, beach land, urban land, rural residential area, other built-up areas, and other unused land). The DEM data were derived from the geospatial data cloud [52]; the data type was raster data with a resolution of 250 m. Data for roads and rivers were derived from the Resources and Environment Science and Data Center of the Chinese Academy of Sciences [51]; the data type was vector data. Road data included data from national highways, railways, and highways. River data had a spatial distribution of 1–5 rivers.

## 3. Research Method

### 3.1. Technical Route

**Figure 2:**
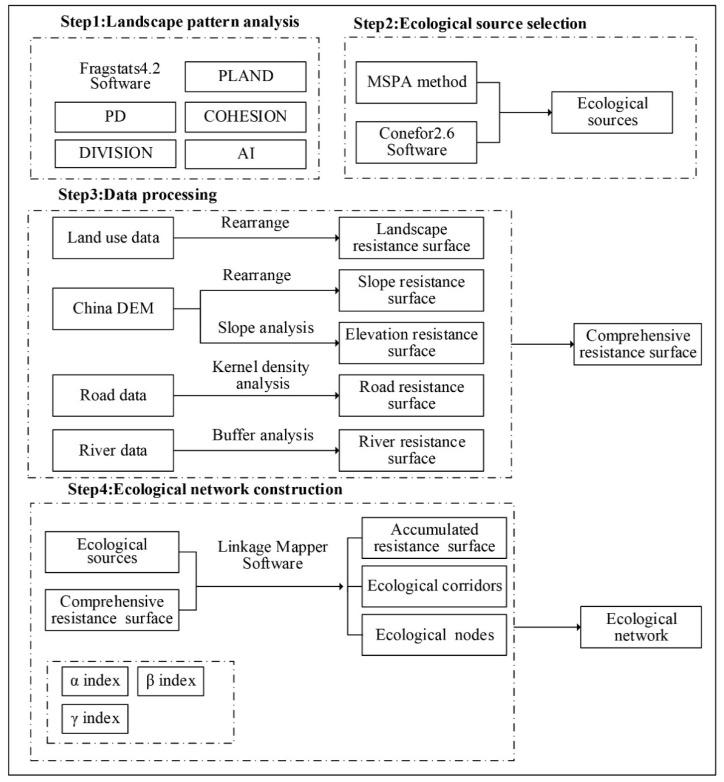
Technical route. Notes: PLAND (Percentage of Landscape) refers to landscape percentage, PD (Patch Density) refers to patch density, COHESION (Patch Cohesion Index) refers to the cohesion index, DIVISION (Landscape Division Index) refers to the landscape separation index, AI (Aggregation Index) refers to the aggregation index. α index refers to the network closure index, β index refers to the network connectivity index, γ index refers to the network connectivity rate.

The study was divided into four steps (Figure 2). First, based on the landscape pattern analysis method, we used the Fragstats4.2 software (Oregon State University, Corvallis, OR, USA) to calculate the index values of PLAND (Percentage of Landscape), PD (Patch Density), COHESION (Patch Cohesion Index), DIVISION (Landscape Division Index), and AI (Aggregation Index) in Chongqing to analyze the evolution process of land use in Chongqing from 2005 to 2015. Second, using land use data from 2015, applying the MSPA method and the Conefor2.6 software (Jenness Enterprises, Flagstaff, AZ, USA), the ecological sources of Chongqing were selected. Then, based on available land use data, DEM data, road data, and river data, the appropriate GIS method was applied to obtain the resistance value, determine the landscape resistance surface, and generate the comprehensive resistance surface with reference to previous studies and specific conditions in Chongqing. Finally, we used the Linkage Mapper software to input the obtained ecological source data and the comprehensive resistance surface data to generate the ecological corridor and the accumulated resistance surface. By overlaying ecological sources, ecological corridors, and ecological nodes, Chongqing’s ecological network was finally constructed. According to the number of ecological corridors and nodes, the ecological network was evaluated and analyzed using the α index, β index, and γ index.

### 3.2. Landscape Pattern Index Analysis

The landscape pattern index is a quantitative method that provides different landscape indices for streamlining the structure of the landscape spatial pattern and the concise landscape spatial morphological characteristics [53]. Taking into account the specific ecological situation of Chongqing and other relevant data [54,55], we selected five landscape indices, namely PLAND, PD, COHESION, DIVISION, and AI, to depict the landscape characteristics of Chongqing. Among them, PLAND quantifies the proportion of each patch type area in the landscape; the larger the proportion, the larger the corresponding patch type area. The Patch Density represents the degree of landscape fragmentation; the greater the value, the higher the fragmentation degree. The Patch Cohesion Index indicates the degree of physical connection between patch types; greater values indicate better landscape connectivity. The Landscape Division Index reflects cuts of the landscape by urban roads and other factors, with greater values indicating that cutting is more obvious and landscape fragmentation is high. The Aggregation Index reflects the connectivity inside the patch; the larger the value, the better the connectivity. Related work has been completed using the Fragstats4.2 software [56].

### 3.3. Selection of Ecological Sources

#### 3.3.1. Identification of Core Ecological Patches by the MSPA Method

The MSPA method originates from mathematical morphology and was initially applied in studies on forest fragmentation [57,58]. When Peter Vogt developed the Guidos software (European Commission Joint Research Centre, Ispra, Italy), MSPA was formally applied to the research of landscape connectivity [59]. By using the ArcGIS software (Environmental Systems Research Institute, Redlands, CA, USA), the land use type data in raster data format were divided into raster binary maps of foreground and background. Forestland, grassland, water area, among others, are generally taken as the foreground and other land use types as the background. Using the Guidos Toolbox software, the foreground was divided into the seven categories core area, bridge area, edge area, branch line, isolated island, ring road, and pore [60]. Among them, core areas generally refer to ecological sources, and the bridging zone is a corridor connecting ecological source patches; both have significant impacts on the connectivity of regional ecological landscapes [61].

We conducted MSPA analysis on land use data of Chongqing using the Guidos Toolbox software platform to identify the core ecological patches of Chongqing, with the following steps:

1. Standardized Data Processing

The land use data of Chongqing for 2015 were loaded into the ArcMap, and the forest land, grassland, and water areas were set as the foreground with a value of 2. The other land classes were assigned as the background with a value of 1 and converted into a binary grid map. Finally, the land use data were exported to the TIFF format.

2. Neighborhood Rule Setting

After loading the binary grid map of Chongqing in the Guidos Toolbox software, the neighborhood structure rules of foreground pixels were set. The software provides two kinds of neighborhood structure: the eight-neighborhood structure and the four-neighborhood structure. Structural elements refer to the units that deal with the target landscape map. The selection of structural elements affects the movement law and the osmotic critical threshold of species in habitat patches. Here, we selected the eight-neighborhood structure for MSPA analysis.

3. Edge Width Setting

The width of the ecological corridor has a direct impact on the analysis results for an ecological network. The terrain and other features analyzed in previous studies [62,63] are similar compared to our study. In addition, because the width increases, a small core area becomes an island, and a narrow core area becomes a bridge. At the same time, combined with the actual situation in Chongqing, we selected a 30 m edge width. In the MSPA method, corridor width is equal to the edge width multiplied by the pixel resolution, that is, the edge width can be determined according to the value of the corridor width and the pixel resolution. The raster data pixel size was 1000 × 1000 m, the width of the ecological corridor was set to 30 m, and the corresponding edge width parameter was set to 0.03.

4. Generation of Seven Landscape Types

After importing the binary grid diagram in the Guidos Toolbox software, we set the required parameters. We used MSPA to generate seven landscape types, non-overlapping and independent of each other: core area, island, pore, edge area, bridge area, ring road, and branch line.

#### 3.3.2. Identification of Ecological Sources by the Landscape Connectivity Index

The MSPA method can identify regional core ecological patches, but it cannot distinguish their importance. This paper uses the landscape connectivity index to classify the importance of the identified ecological patches in the core area [64], and the larger area and higher connectivity in the patches in the core area were selected as ecological sources. Currently, the commonly used landscape connectivity index includes overall connectivity (*IIC*, Equation (1)), possible connectivity (*PC*, Equation (2)), and plaque importance (*dPC*, Equation (3)) [65]. We therefore selected the overall connectivity index, *IIC*, and the possible connectivity index, *PC*, to calculate the patch importance value, *dPC* and, consequently, to evaluate the relative importance of ecological connectivity in the core area. We used equations described elsewhere [66]. The overall connectivity index was calculated as

(1) $$IIC=\frac{\sum_{i=1}^{n}\sum_{j=1}^{n}\frac{{}_{a_{i}}{}^{\times }{}_{a_{j}}}{1+{nl}_{ij}}}{{A_{l}}^{2}}$$

where 0 < *IIC* < 1; when *IIC* equals 1, all landscapes are occupied by habitats. Factors. *a_i_* and *a_j_* refer to the area of patch “*i*” and patch “*j*”, respectively, and *nl_ij_* is the number of connections between patch “*i*” and patch “*j*”. *A_l_* represents the total landscape area of the study area. The *PC* was calculated as

(2) $$PC=\frac{\sum_{i=1}^{n}\sum_{j=1}^{n}a_{i}\times a_{j}\times {P^{*}}_{ij}}{{A_{l}}^{2}}$$

where $p_{ij}^{*}$ refers to the maximum connection probability of two patches. The calculation result of *PC* ranges between 0 and 1, and the *PC* value represents the high or low possibility of landscape connection. $p_{ij}^{*}$ represents the maximum possible connectivity probability between patch “*i*” and patch “*j*”. In general, the smaller the distance between patches, the higher the probability of maximum possible connectivity will be and vice versa. The *dPC* was calculated as follows:

(3) $$dPC=\frac{PC-{PC}^{\prime }}{PC}\times 100\%$$

where the *dPC* refers to the important value of the possible connectivity index of the patch, *PC* refers to the possible connectivity index of a patch in the landscape, and *PC*′ refers to the possible connectivity index of the landscape after removing the patch. For example, when *PC* is 80 and *PC*′ is 20, the calculated *dPC* is 75.

We calculated *dPC* in the Conefor2.6 software. First, the Conefor Toolbox plug-in in ArcGIS was used to extract the distance information between the core patches generated by MSPA, and subsequently, we entered the generated file into the Conefor2.6 software to solve the important value of connectivity. According to the geography of Chongqing, and based on relevant literature [25], the threshold of connectivity distance was set as 500 m, and the connectivity probability was 0.5. According to relevant studies [61], when *dPC* > 1, the patch’s connectivity is better and the patch is more important. Therefore, the core patch of the case where *dPC* > 1 was selected as the ecological source in this paper.

### 3.4. Resistance Surface Construction

Different types of patches will cause different resistances to species migration. The magnitude of resistance can reflect the difficulty of species migration. For example, the resistance value of forest land and green land is small, which is conducive to species migration, whereas that of cultivated land, built-up areas, and unused land is larger, impeding species migration. In addition, resistance factors such as elevation, slope, roads, and rivers also affect species migration. Based on the actual situation in Chongqing, we comprehensively considered the influencing factors of land use types, elevation, slope, roads, and rivers and finally determined the resistance value of various landscape patches in Chongqing (Table 1), based on relevant studies [25,37].

#### 3.4.1. Construction of Landscape Resistance Surfaces

Based on the GIS software platform and the characteristics of Chongqing, we converted the land use raster data for 2015 into vector data and assigned corresponding resistance values according to different types, which were finally converted into landscape type raster data to generate the landscape resistance surface. The DEM data of Chongqing were reclassified and assigned to generate the elevation resistance surface. We then performed slope analysis and reclassification of Chongqing DEM data and generated the slope resistance surface after assignment. The core density of road element data in Chongqing was calculated, and the corresponding search radius parameters were set to generate the road resistance surface. The river data of Chongqing were graded and assigned, and the river resistance surface was generated. All landscape resistance values are shown in Table 1.

#### 3.4.2. Construction of the Integrated Resistance Surface

Each resistance surface contributes to the comprehensive resistance surface according to a certain weight, making it necessary to adequately allocate the weight of each resistance surface before constructing the comprehensive resistance. In this paper, we adopted the analytic hierarchy process [12] to determine the weight of each resistance surface, in combination with the opinions of relevant experts. The greater the weight given, the higher the importance. Based on this, the weights of a total of five resistance surfaces of the landscape type resistance surface, elevation resistance surface, slope resistance surface, road resistance surface, and river resistance surface were set to 0.30, 0.10, 0.10, 0.25, and 0.25, respectively (Table 1). The integrated resistance surface was obtained by weighted superposition of the grid calculator tool of the ArcMap software as the cost data of the minimum cost distance model.

#### 3.4.3. Construction of the Cumulative Resistance Surface

The cumulative resistance surface was generated using the Linkage Mapper software tool in ArcGIS. This tool facilitates the analysis of the connectivity of regional animal habitat [68] and identifies and maps the lowest-cost relationship among ecological sources through ecological source vector data and integrated resistance surface raster data. Each pixel of the integrated resistance surface has a value that reflects the difficulty of the species passing through that pixel. It is usually the pixel feature that determines the resistance value, such as land use type or elevation, slope, road, river, among others. The cost-weighted distance will produce a cumulative resistance surface when the species leaves a specific ecological source area.

### 3.5. Minimal Cumulative Resistance Model

The *MCR* model, first proposed by Knaapen et al. in 1992, is used to simulate the minimum path of species passing through different types of spatial resistance from ecological sources [69]. The model has been improved to identify the ecological corridor [70]. The equation is as follows [71]:

(4) $$MCR=f_{\min}\sum_{j=n}^{i=m}\left(D_{ij}\times R_{i}\right)$$

where *MCR* is the minimum cumulative resistance, “*f*” is the positive function relationship between the minimum cumulative resistance and the ecological process, “min” is the minimum cumulative resistance of the evaluated patches to different sources, “∑” is the cumulative value of the distance and resistance between landscape unit “*i*” and ecological source patch “*j*” across all units, “*D_ij_*” is the spatial distance of species from landscape unit “*i*” to ecological source patch “*j*”, and “*R_i_*” represents the resistance coefficient of the landscape unit “*i*” to the movement of a certain species.

### 3.6. Evaluation of the Ecological Network Index

An ecological network is formed by connecting ecological sources with corridors and various ecological function nodes, and its quality can be evaluated via landscape connectivity. In this paper, we selected the network closure index (α index), the network connectivity index (β index), and the network connectivity rate (γ index) to analyze the ecological corridor network structure and to analyze and evaluate the network closure and connectivity of the Chongqing ecological network [72], with the aim of quantifying the landscape connectivity and complexity of Chongqing. It can not only provide a scientific basis for the construction of the Chongqing ecological network but also represents a reference for the further optimization of this network. The equations for the above three indices are as follows [73]:

(5) $$\alpha =\frac{L-V+1}{2V-5}$$

(6) $$\beta =\frac{L}{V}$$

(7) $$\gamma =\frac{L}{3\;(V-2)}$$

where “*L*” represents the number of corridors, “*V*” is the number of nodes, “*L* − *V* + 1” is the actual number of loops, “2*V* − 5” is the maximum number of possible loops, and “3 (*V* − 2)” represents the maximum number of possible corridors in the network.

## 4. Results and Discussion

### 4.1. Landscape Pattern Index

**Figure 3:**
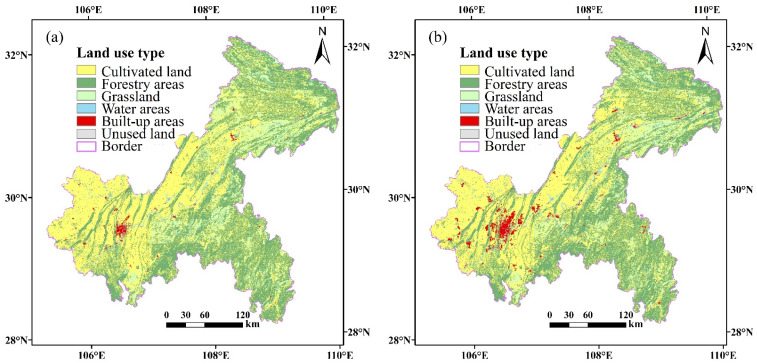
Land use types in Chongqing in 2005 (**a**) and 2015 (**b**).

According to the land use data of Chongqing for 2005 and 2015 (Figure 3a,b), the values of each landscape pattern index in Chongqing were obtained (Table 2). Furthermore, the landscape pattern index was evaluated and compared, and the evolution trend of land use types in Chongqing from 2005 to 2015 was obtained.

Among the land use types of Chongqing in 2005 (Table 2), the proportions of cultivated land and forested land both exceeded 40%, and the proportion of unused land was lowest with only 0.02%. This indicates that the cultivated land area and the forested area in Chongqing were larger in 2005. The patch density of cultivated land and forestry areas in Chongqing was higher, and the patch density of the water area and unused land was lower, indicating a high degree of fragmentation for cultivated and forest areas and a low degree for water areas and unused land. The patch cohesion index of all landscape types was above 90, and landscape connectivity was high. For cultivated and forested land, the landscape separation degree was 0.98, and that of other landscape types was 1, indicating serious landscape segmentation. The aggregation index of unused land was 87.19 and that of other landscape types was above 90, indicating a high connectivity, except for unused land.

Compared to 2005, in 2015, the proportions of forest land area, water area, and built-up areas were higher. This increase was most significant for built-up areas with 1.5 percentage points, reflecting the large-scale expansion of built-up areas in the process of urbanization. The proportions of cultivated land, grassland, and unused land were lower, with the greatest decline for grassland (1.46%); the cultivated land area only decreased by 0.88%. The cultivated land area in Chongqing was 0.01 n/ha lower in 2015 than in 2005, whereas the built-up area was 0.02 n/ha higher. The patch density of other landscape types did not change considerably, reflecting the occupation and destruction of cultivated land in the process of urbanization in Chongqing over 10 years. The patch cohesion index values of cultivated land, water area, and built-up areas increased slightly, indicating that the connectivity of cultivated land, water area, and built-up areas in Chongqing was high during this period. Affected by human activities, the connectivity of grassland and unused land reduced. When comparing the landscape separation index of each landscape type, the division of landscape types in Chongqing was not obvious during the 10 years. Except for grassland and unused land, the aggregation index values of the other landscape types increased, which was most significant for the built-up areas.

When comparing the land use types in 2005 and 2015, cultivated land was the dominant landscape type in both years. Cultivated land fragmentation increased over time, and the connectivity between forested areas decreased. As the ecological network has the function of repairing the ecosystem, these changes show that the urgency of constructing the ecological network in Chongqing is more apparent.

### 4.2. Ecological Source Selection

#### 4.2.1. Identification of Core Ecological Patches

**Figure 4:**
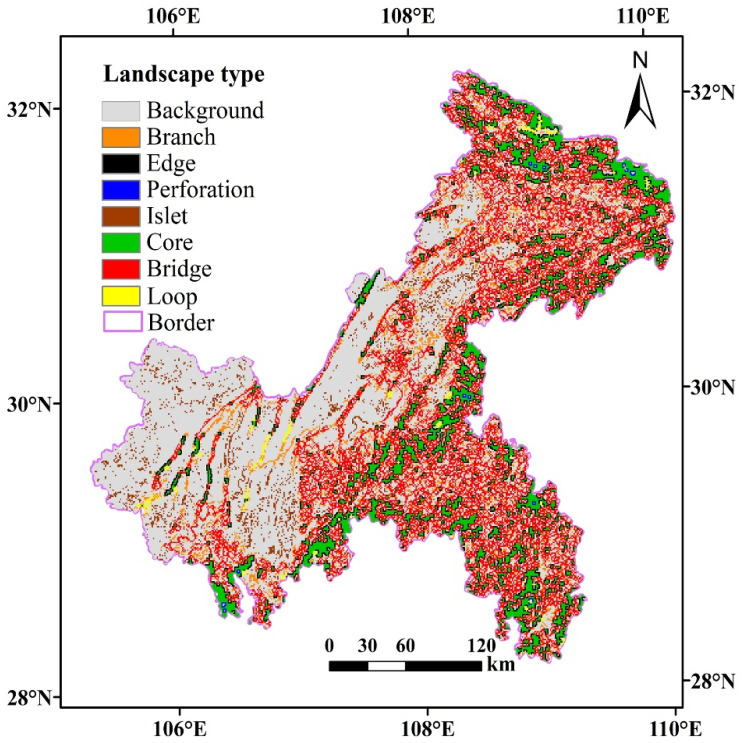
Landscape type map of Chongqing generated via Morphological Spatial Pattern Analysis.

Based on the generated binary grid, the foreground area was 42,973 km^2^, accounting for 52.03% of the total landscape area. The MSPA showed that, for the seven landscape types generated (Figure 4), the proportions of all kinds of landscapes followed the order bridge (50.69%), core area (16.70%), edge (14.35%), branch (11.36%), isolated island (5.68%), loop (1.02%), and perforation (0.20%). Among them, the bridge area is the current corridor in the region, which is conducive to the spread of species and to energy flows. The area of the bridge area was 21,781 km^2^, accounting for 50.69% of the total foreground area. The number of ecological corridors in Chongqing is larger, facilitating the construction of ecological corridors in Chongqing. The core area, which plays a key role in the level of ecological network connectivity, has a large scale, with an area of 7177 km^2^, accounting for 16.70% of the total area of the foreground land category. Compared with other related studies [65,74], the core area is relatively small, most likely because of the specific natural geographical conditions of Chongqing, with a more fragmented landscape and a smaller core area. However, the bridge area of Chongqing is larger than that in other cities, which is more conducive to the construction of ecological corridors.

In addition, the patches of core area are unevenly distributed, with most of its area being in the northeastern and the southeastern parts of Chongqing. Most likely, this is because of the large nature reserves such as Daba Mountain, Yintiaoling Mountain, and Wushan in the northeast and the large landscapes such as Jinfo Mountain, Black Valley, and Qingxi Gou Reservoir in the southeast. The edge area is the transition area between the core area and the background landscape and can reduce the interference of external factors, with an area of 6167 km^2^, accounting for 14.35% of the total foreground area. The branch also has a certain connectivity, with an area of 4881 km^2^, accounting for 11.36% of the total foreground area. The isolated islands are mainly small patches distributed inside the building land, with an area of 2441 km^2^, accounting for 5.68% of the total foreground area. The loop is a shortcut for species movement within the same core area, covering an area of 438 km^2^ and accounting for 1.02% of the total foreground area. The perforation with the same edge effect is the inner edge of the core area, with an area of only 88 km^2^, accounting for 0.20% of the prospects. In general, Chongqing fulfills the requirements for the construction of an ecological network, and further analyses of the core area patches are crucial to screen out ecological sources.

#### 4.2.2. Identification of Ecological Sources

**Figure 5:**
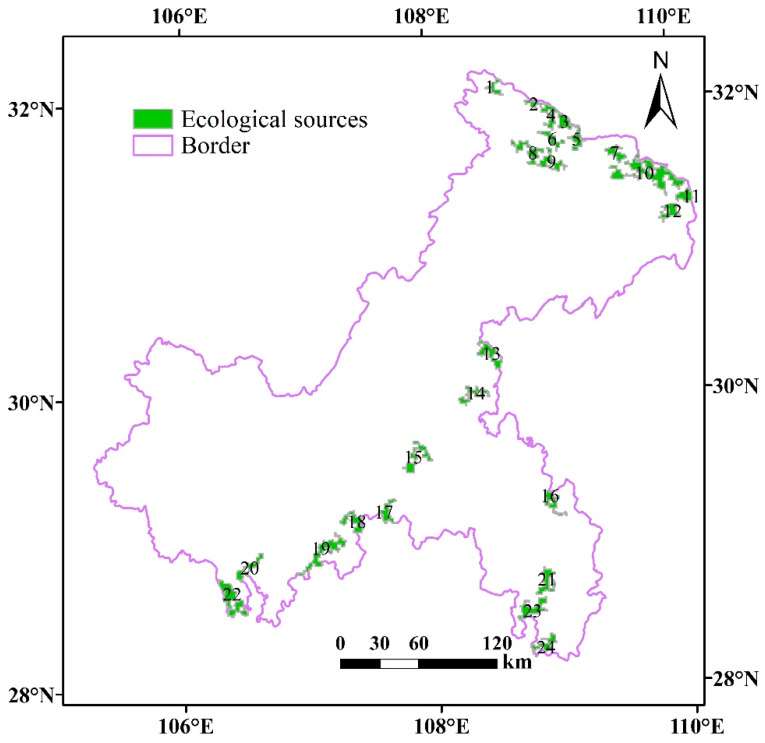
Distribution of ecological sources in Chongqing.

The important value of connectivity (*dPC*) of the core patch generated by MSPA shows that the greater the *dPC* values of the core patch, the greater the contribution of the patch to the overall landscape connectivity. The ecological network constructed in this paper should not only play a macro-role in maintaining regional ecological security, but also guide the layout of land use in ecological construction. Overall, we obtained 24 source plots (Figure 5).

We selected 24 core patches with high connectivity as ecological sources. These patches not only have large area but can also protect the biodiversity and strengthen the landscape connectivity. The *IIC* value, the *PC* value, and *dPC* value of the 24 ecological sources were calculated according to Equations (1)–(3); the total area was 2961 km^2^, accounting for 68.90% of the total foreground area. The largest ecological source was source No. 10, with an area of 447 km^2^. The *IIC* and *PC* values were also the largest, indicating that the connectivity between them and other ecological sources is the strongest and plays a key role in the ecological process.

### 4.3. Resistance Surface Analysis

**Figure 6:**
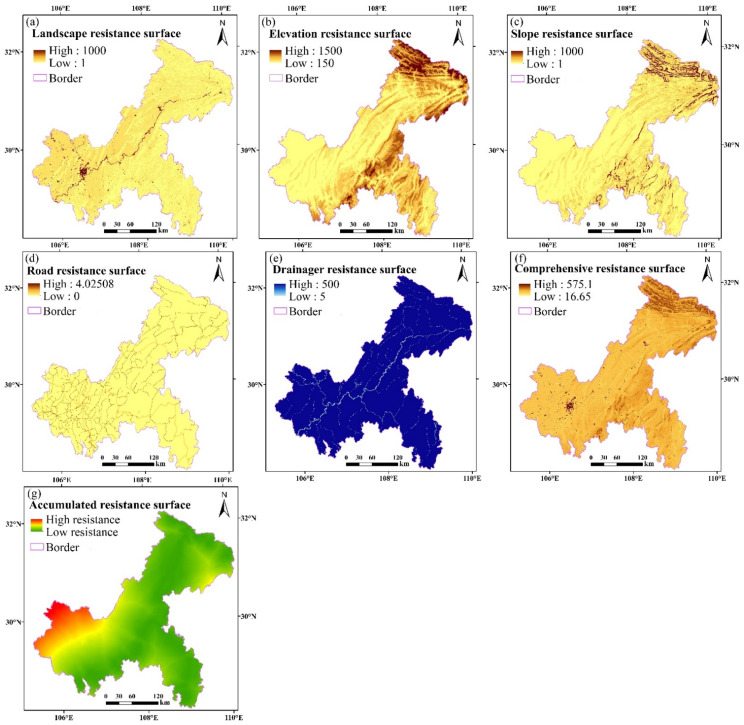
Landscape resistance surface (**a**), elevation resistance surface (**b**), slope resistance surface (**c**), road resistance surface (**d**), river resistance surface (**e**), comprehensive resistance surface (**f**), and cumulative resistance surface (**g**) obtained for Chongqing.

Based on all data types for Chongqing, by the construction method of each front resistance surface, the landscape resistance surface map, the comprehensive resistance surface map, and the accumulative resistance surface map were obtained (Figure 6), and analysis and evaluation were carried out.

Based on Figure 6a, the landscape types of Chongqing are rich and diverse. The resistance value of the western main urban area is high, and the resistance value of species movement in this area is high, followed by the central part of the area and the northeast of Chongqing. The resistance values of other areas with rich forest and grassland resources are low, and the resistance value of species movement or migration in these areas is also low. The spatial distribution of elevation and slope resistance values in Chongqing is similar. The resistance values in the northeast and southeast of Chongqing are higher, along with those of species migration, whereas the values for other areas are relatively low, indicating easier species migration. Based on the road resistance surface (Figure 6d), the resistance value is higher in the southwest of Chongqing, where the road network is dense and it is difficult for species to move or migrate, whereas the resistance value in the southeast is smaller and the road network is less distributed, which is conducive to species movement or migration. According to the river resistance surface, in the southeast, species movement and migration are easier. Finally, according to the comprehensive resistance surface (Figure 6f), the resistance values of the southwestern and northern parts of Chongqing are larger, impeding species movement.

In general, the accumulative resistance surface of Chongqing is lower in the south and east, higher in the southwest, and unevenly distributed in the north. Considering that, generally, species choose paths with lower resistance for movement and migration, the cumulative resistance surface provides a data basis for the construction of an ecological networks.

### 4.4. Construction of Potential Ecological Corridors in Chongqing

#### 4.4.1. Identification of Potential Ecological Corridors in Chongqing

**Figure 7:**
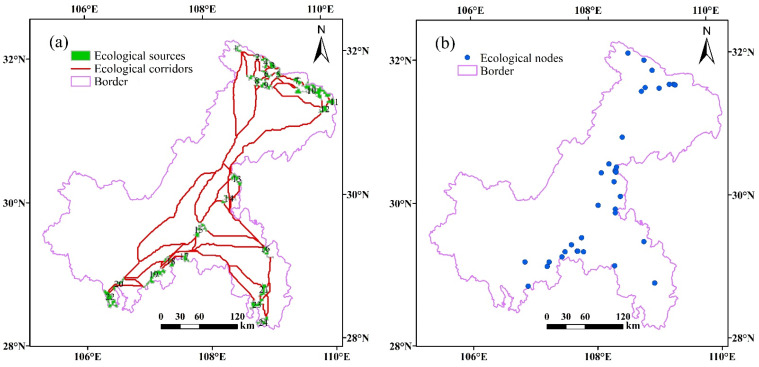
Potential ecological corridor (**a**) and ecological node distribution (**b**) in Chongqing.

Based on the ArcGIS software, the corresponding parameters were set up by the Linkage Mapper software to construct the potential ecological corridors of Chongqing (Figure 7a). Overall, there are 87 potential corridors in Chongqing, with a total length of 2524.34 km and an average length of 29.02 km. Spatially, the ecological corridors are mainly concentrated in the northern and southeastern areas, connecting 24 ecological patches.

#### 4.4.2. Ecological Node Identification

Ecological node quantity, quality, and spatial distribution affect the efficiency of species activity or migration. The results of ecological node extraction in Chongqing are shown in Figure 7b above. There are 35 ecological nodes in Chongqing, mainly distributed in the northeast and south, with an uneven distribution. Therefore, based on the particular geographical location of Chongqing, it is necessary to improve the stability of regional ecological nodes and promote their coordination, ensuring that each node can adequately be protected and restored.

### 4.5. Construction of Chongqing’s Ecological Network

#### 4.5.1. Identification of Chongqing’s Ecological Network

**Figure 8:**
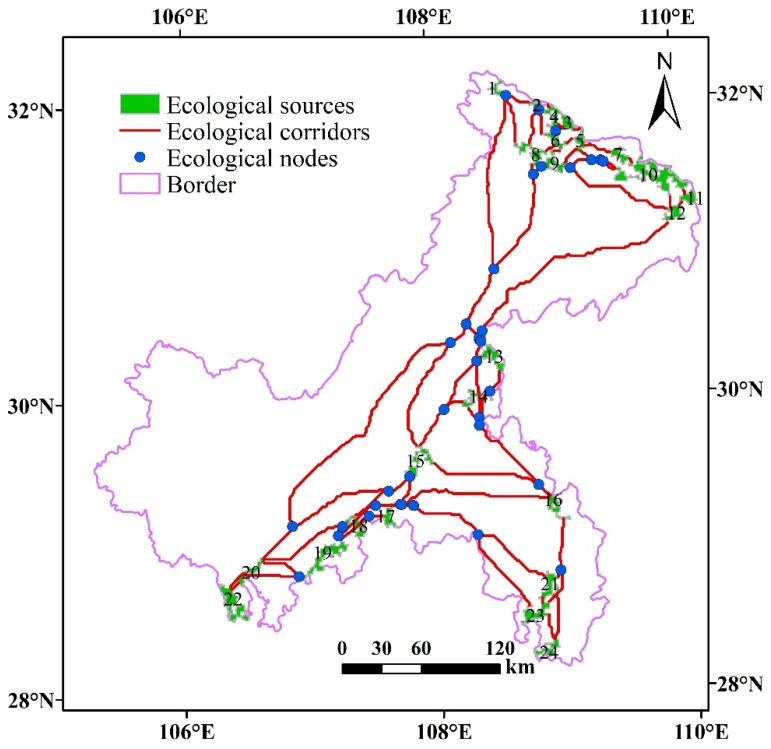
Chongqing ecological network.

The ecological network of Chongqing was constructed by overlaying ecological sources, ecological corridors, and ecological nodes (Figure 8). The distribution of ecological corridors and ecological nodes in the north and southeast of Chongqing is relatively dense, and the large core areas in the southwest consist of only a few patches. The rapid urbanization of Chongqing has brought great pressure to the ecological environment, and the landscape is more fragmented. The ecological network provides the best path choice for species movement or migration, avoiding risks and maintaining biodiversity to a large extent.

The unique geographical conditions of mountainous areas challenge species survival and migration. However, with the development of urbanization worldwide, the destruction of mountainous ecosystems is unstoppable [73]. As a country with a significant amount of mountainous terrain, China needs to pay more attention to the protection of mountain environments. In recent years, the deterioration of the ecological environment in Chongqing has also attracted the attention of the Chinese government and scholars [75]. The establishment of ecological networks as an effective means to maintain biodiversity should also be considered. In addition, the construction of ecological networks requires financial and material support. On the basis of existing nature reserves, the Chinese government should increase investments in the construction of ecological networks in Chongqing, especially in the southwest, and the following measures should be taken: generating urban green spaces and other green landscapes, formulating relevant policies, strictly managing urban industrial development, and scientifically planning built-up areas.

#### 4.5.2. Ecological Network Index Analysis

The ecological network in Chongqing contains 87 ecological corridors and 24 ecological sources. By calculating the α index, the β index, and the γ index, the structural characteristic index of the ecological network in the study area was obtained (Table 3), and the constructed ecological network was evaluated and analyzed.

The value of the α index ranges from 0 to 1, with larger values indicating a higher number of loops in the network, a higher accessibility of the ecological flows in the network, and a higher number of paths that can be selected for migration. For the ecological network constructed in this paper, the actual number of loops is 53, the maximum possible number of loops is 65, and the value of the α index is 0.82, indicating a high connectivity of the network. Species flow or migration and other ecological processes are extremely smooth. However, the distribution of network circuits in Chongqing is uneven, and the network circuits composed of ecological sources and ecological corridors in the central and southwestern regions are less. The construction of ecological sources needs to be increased in this part of the region to strengthen the network circuits, providing more path choices for species migration.

The value of the β index ranges between 0 and 3; the larger the value, the higher the complexity of the network. Here, the β index is 2.49, which indicates that the ecological network is more complex as a whole, and the interaction among ecological sources in the network is facilitated.

The value of the γ index ranges between 0 and 1, and the larger the value, the higher the connection degree of the ecological sources. In this paper, this value is 0.89, indicating that the ecological corridor in the ecological network connects more ecological sources and that the network efficiency is better. However, due to the small number of ecological sources and ecological corridors in the southwest of Chongqing, species movement and migration are impeded. Therefore, to promote the diffusion of ecological flows in Chongqing and improve biodiversity protection, corridor connection in the southwest should be strengthened.

## 5. Conclusions

Based on land use data for Chongqing, China, from 2005 and 2015, we used MSPA and the Conefor2.6 software to identify 24 important ecological sources. Based on the *MCR* models and the Linkage Mapper software, 87 potential ecological corridors and 35 ecological nodes were constructed. Finally, the ecological sources, corridors, and nodes were superimposed, and Chongqing ecological network was generated. The total length of the potential ecological corridor is 2524.34 km, with an average corridor length off 29.02 km. The α index, β index, and γ index values indicate a good connectivity between the selected ecological sources, with a high network efficiency.

Against the background of topography and urbanization, the number of ecological sources in Chongqing is small and the spatial distribution is uneven. They are mainly distributed in the northeast and southeast, with fewer sources in the southwest. The spatial distribution of ecological nodes is similar to that of ecological sources. The spatial distribution of potential ecological corridors is not balanced, as they are concentrated in the northeast and southeast, indicating a poor efficiency of the ecological network.

Our results provide a scientific basis for the protection of Chongqing’s ecological environment. Regarding the southwestern part of Chongqing, it is necessary to strengthen the connectivity between the sources, thereby further improving the ecological network. In addition, the parameter setting of the resistance value is relatively subjective. Based on the current land use data for Chongqing, economic factors should be considered, and the effective resistance surface data for landscape, elevation, slope, roads, and rivers should be further combined.

## Figures and Tables

**Table 1:** Resistance values and weights of resistance factors in Chongqing.

Resistance Factors	Weight	Classification Indicators	Resistance Value
Land use types	0.30	Forests	1
		Shrubs, sparse forests	3
		Paddy fields, dry lands	50
		Other forestry areas	300
		High-coverage grassland	10
		Medium-cover grassland	15
		Low-coverage grassland	20
		Rivers	600
		Lakes	300
		Reservoir Pit Tong	100
		Beach	1
		Urban land, rural settlements	900
		Other construction sites	1000
		Others	700
Elevation	0.10	Elevation range	Resistance value
		<450 m	150
		450–700 m	300
		700–1000 m	500
		1000–1400 m	800
		1400–1800 m	1000
		>1800 m	1500
Slope	0.10	Slope range	Resistance value
		<3°	1
		3–6°	20
		6–10°	100
		10–16°	200
		>16°	600
Roads	0.25	Road types	Resistance value
		Railways	700
		National Highway	2000
		Other roads	500
Rivers	0.25	Buffer	Resistance value
Level I rivers		<50 m	5
		50–120 m	25
		120–300 m	50
Level II rivers		<50 m	10
		50–120 m	50
		120–300 m	100
Level III rivers		<50 m	20
		50–120 m	100
		120–300 m	500

Note: Resistance value data in Table 1 are derived from literature [39,42,67].

**Table 2:** Calculation results of the landscape pattern index in Chongqing for 2005 and 2015.

Landscape Index	Year	Cultivated Land	Forestland	Grassland	Water Areas	Built-Up Areas	Unused Land
PLAND (100%)	2005	46.49	40.45	11.00	1.16	0.88	0.02
	2015	45.61	41.01	9.54	1.45	2.38	0.01
PD (n/ha)	2005	0.31	0.15	0.08	0.01	0.02	0.00
	2015	0.30	0.15	0.08	0.01	0.04	0.00
COHESION	2005	99.83	99.95	99.47	99.77	98.32	93.23
	2015	99.84	99.95	99.10	99.81	98.84	93.01
DIVISION	2005	0.98	0.98	1.00	1.00	1.00	1.00
	2015	0.98	0.97	1.00	1.00	1.00	1.00
AI	2005	94.84	95.08	92.66	93.73	93.34	87.19
	2015	94.87	95.15	92.59	94.09	95.21	86.64

Notes: “PLAND” represents landscape percentage; “PD” represents patch density; “COHESION” represents the cohesion index; “DIVISION” represents the landscape separation index; “AI” represents the aggregation index; “n/ha” represents the number of landscape patches per 100 hectares.

**Table 3:** Characteristics of the Chongqing ecological network.

	Index	L	V	L − V + 1	2V − 5	3 (V − 2)	Results
Ecological Network	α index	87	35	53	65		0.82
	β index	87	35				2.49
	γ index	87	35			99	0.89

## Data Availability

All relevant data sets in this study are described in the manuscript.
